# Polyploidization in Orchids: From Cellular Changes to Breeding Applications

**DOI:** 10.3390/plants11040469

**Published:** 2022-02-09

**Authors:** Joe Abdul Vilcherrez-Atoche, Carla Midori Iiyama, Jean Carlos Cardoso

**Affiliations:** 1 Master Science Graduate Program of Plant Production and Associated Bioprocesses, Center of Agricultural Sciences, Federal University of São Carlos, Araras 13600-970, SP, Brazil; carlaiiyama@gmail.com; 2 Laboratory of Plant Physiology and Tissue Culture, Department of Biotechnology, Plant and Animal Production, Center of Agricultural Sciences, Federal University of São Carlos, Araras 13600-970, SP, Brazil

**Keywords:** floriculture, *Orchidaceae*, breeding, polyploidy, hybridization, colchicine

## Abstract

Polyploidy occurs naturally in plants through cell division errors or can artificially be induced by antimitotic agents and has ecological effects on species adaptation, evolution, and development. In agriculture, polyploidy provides economically improved cultivars. Furthermore, the artificial induction of polyploids increases the frequency; thus, it accelerates obtaining polyploid plants used in breeding programs. This is the reason for its use in developing many crops of economic interest, as is the case of orchids in the flower market. Polyploidy in ornamental plants is mainly associated with flowers of larger size, fragrance, and more intense coloring when compared to naturally diploid plants. Currently, orchids represent the largest flower market worldwide; thus, breeding programs aim to obtain flowers with the larger size, durability, intense colors, and resistance to pathogens. Furthermore, orchid hybridization with polyploidy induction has been used to produce improved hybrid cultivars. Thus, the objective of this review was to compile information regarding the natural occurrence, importance, and methods of induction of polyploidy in orchids. The study also summarizes the significance of polyploids and techniques associated with artificially inducing polyploidy in different orchids of commercial relevance.

## 1. Introduction

Polyploidy is defined as the increase in chromosome number, generating organisms with more than two complete sets of chromosomes. Polyploidy is one of the essential phenomena in plants and is responsible for species adaptation, diversification, evolution, and development [1]. It is estimated that about 70% of angiosperms have experienced polyploidy during their evolutionary history [2]. The highest frequencies of duplication of genetic material were mainly observed in domesticated plants instead of wild plants. The close relationship between domestication and polyploidy is due to the random selection of polyploid plants for their greater vigor; thus, polyploid species could be more successful and promising for domestication than wild ones [3]. Throughout evolution, the angiosperm genome experienced at least one chromosome duplication event [4], thus allowing original diploid individuals to generate other plants with different ploidy levels.

Polyploid organisms can be classified according to their origin into autopolyploid and allopolyploid [5]. An autopolyploid organism increases its basic number of chromosomes and is formed by the duplication of its genome [5,6]. An allopolyploid is formed from hybridization between different species and is characterized by having more than two basic sets of different chromosomes [6]. It is believed that the majority of flowering plants are allopolyploids (about 75%) [7,8].

Natural polyploidization in plant cells is often undergone through the endoreduplication process. However, the occurrence of endopolyploidy levels in plant organs are variable [9], and their frequency may differ due to certain factors like the taxonomic position of the plant, size of the genome, and dissimilarities between individuals of the same family or between varieties or ecotypes of the same species [9,10,11,12].

Another interesting aspect of natural polyploidy is the formation of unreduced gametes (2n). Harlane and De Wet [13] stated that 85 plant genera could produce unreduced gametes (2n), which results in natural polyploidization. Similarly, Franke [14] showed that 31 plant families had unreduced functional gametes. The development of unreduced gametes results in polyploidy in plants by the fusion of unreduced gametes or between a reduced and an unreduced gamete, originating polyploid organisms by sexual reproduction. This mechanism is mainly responsible for an increase in chromosome number in plants [5,13].

## 2. The Orchidaceae Family and Its Economic Importance in the World Floriculture

Orchids are a plant family with the largest number of species globally. There are 27,801 species [15], which are distributed worldwide, but their diversity and richness are concentrated in tropical and subtropical regions of the world.

Orchids have high commercial value in the international flower market due to the ornamental characteristics, such as shape, size, color, and durability of their flower [16]. In addition, orchids represent one of the world’s leading markets for cut and potted flowers. The most sought genera of the global floriculture markets are *Phalaenopsis*, *Cymbidium*, *Dendrobium*, and other tropical genera such as *Oncidium*, *Cattleya*, and *Vanda* [17].

Most orchid cultivars used as ornamental crops are the result of crosses between species (interspecific hybrids) and genera (intergeneric hybrids) [16], which give rise to groups of hybrids, some of them are multigeneric [18,19].

Due to the enormous global demand for orchids, many breeding programs have been undertaken for *Phalaenopsis* [20], *Dendrobium* [21], *Cattleya* [22], *Cymbidium* [23], *Oncidium* [24], etc. In addition, their ornamental and horticultural characteristics, such as accelerated development, vegetative and reproductive vigor, hardiness, and early flowering [18], have been incorporated to obtain high-quality new hybrid cultivars.

Among the so-called novelties developed by orchid breeding programs worldwide are the new groups of cultivars of the genus *Phalaenopsis*. These include: the “Harlequins” obtained from *Phal.* “Golden Peoker” through somaclonal variations; and miniaturized cultivars obtained by hybridization, mutation, and transgenics [25,26]. *Phalaenopsis* hybrids have been standing out for some time as the main pot flower in the global flower markets [27,28,29].

Additionally, the induction of polyploids is an important tool in the genetic improvement and hybridization of orchids [30]. This technique has been useful for improving and modifying plants’ vegetative and floral characteristics and also for restoring the fertility of some hybrid progenitors with low or no fertility, such as in triploid plants [31].

## 3. Orchid Karyotype

Cytogenetic knowledge is available only for a small number of orchid species [32]. Most studies have been concentrated on commercially important genera useful in floriculture, medicine, and food condiments, such as *Cattleya*, *Cymbidium*, *Dendrobium*, *Oncidium*, *Phalaenopsis*, *Paphiopedilum*, *Vanilla*, and *Vanda* [32,33,34,35,36,37,38,39]. The most common techniques used for polyploid identification in these orchids are chromosomal counting [38,39,40] and nuclear DNA content estimation using flow cytometry (FCM) [33,34,35,36,37,38,39,40,41].

With 187 species described, most *Cattleya* species have a basic number of chromosomes x = 20. However, there are some exceptions, such as morphological variants of *C. bicolor* (*C. bicolor* ssp. *bicolor* has 2n = 40 and *C. bicolor* spp. *minasgeraensis* has 2n = 80) [32,33,34,35,36,37,38,39,40,41,42], *C. nobilior*, which has 2n = 42, and also other tetraploid species of *Cattleya* (2n = 80) [32]. The other chromosomal variations reported are 21, 27 and 30 [32].

Chromosomal and cytological studies of *Cymbidium* species have demonstrated a predominance of 2n = 40 chromosomes [38,39], with variations observed in *Cym. serratum* (2n = 41, 43, 60, and 80). From the first *Cymbidium* polyploids reported in the early 20th century and through the biological and artificial techniques, it has been possible to develop a set of polyploid cultivars of *Cymbidium* [43]. *Cymbidium* cultivars are diploids, triploids, and tetraploids with reported differences in chromosomal morphology [40]. The polyploids have been reported in 75.8% of *Cym. hybridum* cultivars [44], demonstrating an association between the intentional or nonintentional selection of polyploids instead of diploids for superior characteristics.

In *Dendrobium*, most species have 2n = 38 chromosomes, in some species (e.g., *Den. leonis* and *Den. dixanthum*), they have 2n = 40 [34,37], and eight species of section Lautoria have 2n = 36. In *Dendrobium*, polyploids were associated with some species and hybrids [34].

In *Phalaenopsis*, most species have 2n = 38 chromosomes, except for the section Aphyllae, which has 2n = 34 and 36 chromosomes. However, there was observed a significant variation in the size of the chromosomes and genome of species and hybrids from this genus [36,45]. For example, Aoyama [46] reported significant variations in the number of chromosomes of *Phalaenopsis* cultivars: 2n = 38, 57, and 76–114, demonstrating polyploidy. Lee et al. [36] also observed, in 60 different *Phalaenopsis* hybrid cultivars conventionally used for flower cultivation, a large predominance (80%) of tetraploid cultivars (2n = 70–81 chromosomes) with 55% containing 2n = 4x = 76 chromosomes, with only one diploid (2n = 38) reported.

The high number of commercial tetraploids cultivars demonstrates the importance of polyploidy in the development of superior cultivars of *Phalaenopsis*. In addition, natural tetraploid species, such as *Phal. amabilis* and *Phal. rimestandiana* (both 2n = 4x = 76) and *Phal. aphrodite* subsp. *formosana*, are conventionally used in breeding for the production of tetraploid hybrids [6,20,47]. These tetraploid species are used as parental to obtain different groups of *Phalaenopsis* cultivars with different desired colors for commercial purposes [36,48,49].

Similar to *Dendrobium* and *Phalaenopsis*, orchid species of the genus *Vanda* contains 2n = 38 chromosomes [50,51], and the natural occurrence of tetraploid and hexaploid species [50,51,52]. Interspecific and intergeneric hybrids of *Vanda* with *Aerides*, *Vandopsis*, and *Arachnis* have demonstrated chromosomal uniformity (2n = 38). However, there are irregularities in the meiotic division observed in these intergeneric hybrids [53,54].

In the genus *Oncidium*, the basic number of chromosomes is believed to be x = 7, but unlike other genera, this has a large chromosomal variation among species, with the majority presenting polyploidy, such as tetraploids, hexaploids, and octoploids, numbers of chromosomes [55].

## 4. Natural Occurrence of Polyploid Cells in Orchids

### 4.1. Endopolyploidy

Endopolyploidy, commonly generated by endoreduplication, has been reported in different genera of orchids (Table 1). Tissue type, stage of development (early or late), and differences between varieties within the same species are the main factors that can influence the frequency and intensity of cellular endoreduplication [9,56,57,58]. In addition, abiotic environmental factors such as light and nutrients also affect the endoreduplication in plants [9]. For example, Lee et al. [59] reported that temperature influenced the occurrence of endopolyploidy in the cells of *Phalaenopsis aphrodite* and *Oncidium varicosum* orchids.

Other factors, such as phytoregulators, also influence the presence of different levels of endopolyploidy in plant tissues [9]. Lim and Loh [60] observed that sexual embryos of *Vanda* “Miss Joaquin” in the presence of 1-Naphthaleneacetic Acid (NAA) had higher levels of endopolyploidy in their cells compared to embryos in the presence of Gibberellic Acid (GA_3_), showing that synthetic auxins, such as NAA, are important induction factors that generate variations in ploidy levels in orchid cells.

Several studies have reported endopolyploidy in orchids with a wide variety of cells, tissues, and organs with natural polyploidization events (Table 1).

The main types of plant material used for the analysis and identification of endopolyploidy in orchids were leaf tissues (46% of the works), followed by parts of the flowers and roots. Other types of plant tissues were also reported, such as seeds (9%) and ovarian tissue (9%). Plant tissues from in vitro cultivation have also been used to determine ploidy variations in orchid cells. Protocorms (27%) and protocorm-like bodies (PLBs) (27%) are the most used tissue types to analyze the endopolyploidy, followed by embryos (sexual and somatic) and calluses with 23% and 9%, respectively.

Likewise, different orchid genera have been characterized by different levels of endopolyploidy. For example, nuclei with DNA contents up to 64C were observed in the genera *Doritaenopsis* [61,62] and *Vanda* [60,63], followed by nuclei with DNA content up to 32C for *Dendrobium* [64,65], *Phalaenopsis* [56,57,58,59,66,67,68,69,70], and *Vanilla* [71]. For the genera, *Oncidium* [59,69], *Cymbidium* [58,72,73,74], and *Spathoglottis* [75], nuclei with DNA contents up to 16C were found, and the lowest levels of nuclei with DNA contents were seen for *Cattleya* [76] up to 8C (Table 1).

**Table 1:** Endopolyploidy observed in different orchid species.

Species/Cultivar	Plant Material	Nuclear DNA Content	References
*Cattleya tigrina*	Leaves, leaf bases, leaf tips, roots, Protocorm-like Bodies (PLBs)	2C, 4C, 8C	Liz [76]
*Cymbidium* sp.	Embryo Parenchymal Cells		Nagl [73]
Nine comercial hybrids of *Cymbidium*	Callus and PLBs	2C, 4C, 8C, 16C	Teixeira et al. [58]
*Cym.* Twilight Moon ‘Day Light	Callus and PLBs	2C, 4C, 8C, 16C	Teixeira da Silva; Singh; Tanaka [74]
Two comercial hybrids of *Cymbidium* and *Cym. kanran*	PLBs	2C, 4C, 8C, 16C	Fukai; Hasegawa; Goi [72]
*Dendrobium* sp.	Root tips and new leaves		Jones; Kuehnle [64]
*Den.* Chao Praya Smile	Seeds, Protocorms, Protocorms with leaves, stem tips, axillary buds and pseudobulbs, leaves, roots and flowers	2C, 4C, 8C, 16C, 32C	Seah [65]
*Doritaenopsis* hybrid	Somatic embryos	2C, 4C, 8C, 16C, 32C, 64C	Park; Paek [61]
*Doritaenopsis*	Somatic leaves, roots and embryos	2C, 8C, 16, 64C	Park; Yeung; Paek [62]
*Oncidium varicosum*	Flowers	2C, 4C, 8C, 16C	Lee et al. [69]
*Onc. varicosum*	Flowers	2C, 4C, 8C, 16C	Lee et al. [59]
*Phal. aphrodite* subsp. *formosana*	sepals, petals, lip, columns, pollinia, pedicels, ovaries of fully open flowers, roots, protocorms, seedling leaves	2C, 4C, 8C, 16C, 32C	Chen et al. [66]
*Phal. aphrodite* subsp. *formosana*	Ovarian tissue before/after pollination, seeds and protocorms	2C, 4C, 8C, 16C	Jean et al. [57]
*Phal. aphrodite* subsp. *formosana*	Flowers	2C, 4C, 8C, 16C	Lee et al. [69]
*Phal.* spp.	Protocorms		Chen et al. [56]
*Phal.* spp.	Protocorms, PLBs and young leaves	2C, 4C, 8C, 16C	Chen; Tang; Kao [68]
*Phal.* spp.	PLBs and young leaves	2C, 4C, 8C	Chen; Tang [67]
*Phal.* spp.	Flowers, roots and leaves	2C, 4C, 8C, 16C	Lin et al. [70]
*Spatoglottis plicata*	Leaves, roots, floral tissue, protocorms, young seedling leaves, roots	2C, 4C, 8C, 16C	Yang; Loh [75]
*Vanda* Miss Joaquin	Leaves, buds, aerial and terrestrial roots, petals, sepals, pedicels, spine, sexual embryos	2C, 4C, 8C, 16C, 32C, 64C	Lim; Loh [60]
*V. sanderiana*	Somatic embryos	2C, 4C, 8C	Alvarez [63]
*Vanilla planifolia*	Roots	2C, 4C, 8C, 16C, 32C	Kausch; Horner [71]

PLBs: Protocorm-like bodies.

The endopolyploidy observed in orchid cells of different types of tissues and organs can be used for the induction and regeneration of complete polyploid plants from endopolyploidy cells, thorugh the use of plant tissue culture techniques [56], and that can be usefull as a biotechnological tool for orchid polyploid cultivar development. Chen et al. [56] developed a technique for the genus *Phalaenopsis* that consists of successive cycles of horizontal sectioning of protocorms and PLBs, thus inducing the natural endopolyploidy cells in these organs to new PLBs formation, which formed solid polyploid plants after regeneration [56,67,68].

### 4.2. Occurrence of Unreduced Gametes

In orchids, more than one million pollen grains are grouped into a cohesive mass called pollinia [77]. Cytological pollen studies in orchids have shown the formation of unreduced gametes, which are more frequent in cultivars resulting from interspecific and intergeneric hybridization [78]. In some orchid genera like cultivars of *Cymbidium*, Zeng et al. [79] observed that the frequency of unreduced (2n) gametes ranged from 0.15% to 4.03%, depending on the genotype. After seven different crosses between these cultivars, they observed two tetraploids and three triploid hybrids with good in vitro regeneration behavior and high survival during acclimatization. Thus, progenitor cultivars with a higher frequency of unreduced gametes could be used to induce polyploidy in *Cymbidium* breeding programs without inhibiting the mitotic spindle and with no carcinogenic risk to animal cells, commonly associated with the manipulation and treatment of plant tissues and organs with antimitotic agents, such as colchicine.

The main mechanisms of natural polyploidization in *Phalaenopsis* are hybridization and endopolyploidy. However, after analyzing the chromosomes of 60 *Phalaenopsis* cultivars, Lee et al. [36] suggested that, in addition to endopolyploidy, the formation of unreduced gametes could also be responsible at least in part for the expressive frequency and number of polyploid genotypes.

There are also reports regarding the formation of unreduced gametes in other orchid genera and species, such as *Plocoglottis*, *Calanthe*, *Spathoglotis*, *Phaius* [78], *Bletilla striata* var. *gebine* [80], *Epipactis latifolia* [81], *Aerides odoratum*, *Doritis pulcherrima*, and *Vanda denisoniana* [82].

## 5. Artificial Induction of Polyploidy in Orchids

Polyploidy is artificially induced by applying antimitotic agents such as colchicine, oryzalin, trifluralin, propyzamide, and amiprofos-methyl (APM) on tissues, organs, or entire plants [83]. These chemicals are used in vitro to interfere during cell division, generating chromosome duplication in plant cells [84]. Antimitotic agents are grouped according to the phase of the cell cycle that they affect. Some agents can affect the end of the S phase or middle of the M phase (late-stage). Other agents act before the S phase, being the most significant group used for the artificial induction of polyploids [84]. The substance most widely used for polyploidy induction in plants is colchicine, an alkaloid extracted from the seeds and bulbs of *Colchicum autumnale* plants [84,85]. Before colchicine, Randolph [86] induced artificial polyploidy through high-temperature treatment in early-stage embryos of maize, generating tetraploids. Similarly, Blakeslee and Avery [87] obtained somatic polyploidization using high- and low-temperature heat treatments, but these techniques were not efficient for the induction of polyploids. Blakeslee and Avery [87] and Eigsti [88] conducted the first tests using ex vitro colchicine to plant-inducing polyploidy.

Murashige and Nakano [89] were the first to report spontaneous polyploidy in tobacco callus under in vitro conditions in response to the increase of explant subcultures. They recommended in vitro plant growth as an efficient tool to artificially induce polyploidy [90].

**Figure 1:**
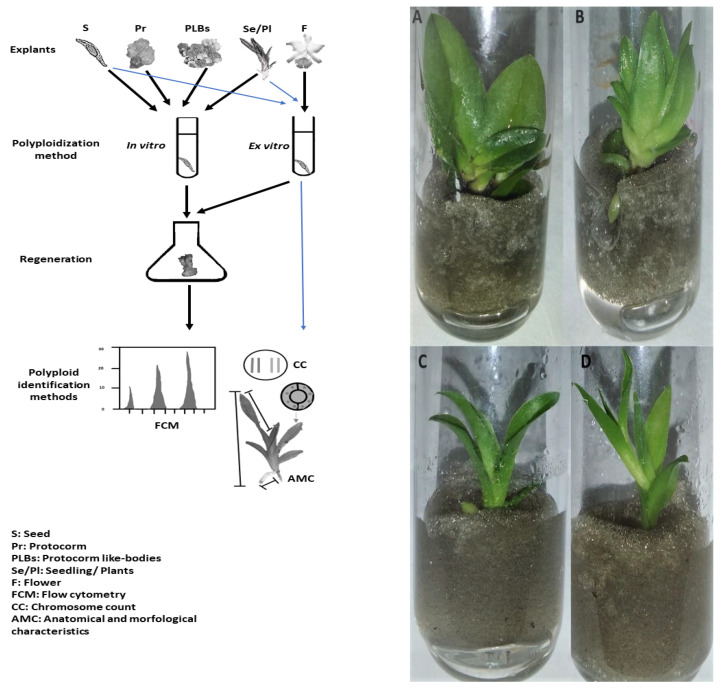
Workflow of the treatment of different types of explants with antimitotic agents aiming to obtain polyploid plants in orchids (left) and morphological differences between autopolyploid plants of hybrid *Cattleya* orchids induced by treatment with colchicine and confirmed by flow cytometry (**A**,**B**) and plants not induced (**C**,**D**).

Currently, there are a large number of protocols for in vitro chromosome duplication in many plant species, including orchids. Figure 1 summarizes the main types of explants used and the workflow aimed at obtaining artificial autopolyploid plants in orchids through chemical antimitotics. The efficiency in generating these types of polyploids depends on the type, concentration, and exposure time to the antimitotic agent, explant type and age, in vitro induction protocol, and direct or indirect methods for confirming chromosomal duplication [84]. Among the various benefits are that polyploidy causes increased vigor, allowing more remarkable adaptation to extreme climatic conditions [91]; an overall increase in organs size due to multiple copies of genes, resulting in a phenomenon known as the gigas effect, is also observed [90,91].

Studies at the Laboratory of Plant Physiology and Tissue Culture (CCA/UFSCar, Araras, Brazil) revealed that in vitro autopolyploid plantlets of the *Cattleya* hybrid induced by colchicine showed distinct morphology. These plants were more compact, with wider and thicker leaves (Figure 1A,B), than those that were not polyploid (Figure 1C,D). Other interesting characteristics associated with polyploid organisms include the buffering genome, heterosis, increased heterozygosity, restoration of hybrid fertility, reduced fertility in autopolyploids, and seedless fruits [89,90]. In addition, flowering in polyploid organisms results in improved ornamental features, such as the larger size and intensity of pigments [92] and longer durability [90]. These characteristics associated with polyploidy are desirable and valuable in orchid breeding programs [93]. Among the various benefits of polyploidy for orchid cultivation, the restoration of fertility of hybrids and changes in the morphological and anatomical characteristics, such as increased leaf thickness and length, increased stomata, and the increased size and texture of flowers, besides influencing the flowering periods, are the most significant ones [94,95].

### 5.1. Cattleya Genus

The induced polyploidy in *Cattleya* can be used for obtaining the compact size of plants, increased flower longevity, a greater number of flowerings throughout the year, flowers with higher firmness (substance), and greater resistance to transport. These are the biggest challenges for the expansion of *Cattleya* cultivation and marketing [18].

In two studies, polyploidy was induced in *Cattleya*, where the PLBs and seedlings were used as explants for in vitro cultivation. Colchicine was used in one study at concentrations of 0.05–0.2%, and the exposure time ranged from two to four days. Another study compared the use of two polyploidy-inducing agents, viz., colchicine (0–12.5 mM) and oryzalin (0–50 µM) (Table 2). Unfortunately, despite numerous hybrids used in the *Cattleya* flower market, only *C. intermedia* and *C. tigrina* have been reported in the literature.

For *C.a intermedia*, the best treatments for polyploid induction using colchicine were 0.05% (for clone 114–75% of tetraploids) and 0.1% (for clone 121–40% of tetraploids), both treated for eight days, showing a strong genotype-dependent response [96].

### 5.2. Cymbidium Genus

The first use of antimitotics and induction of polyploidy in the genus *Cymbidium* was reported by Menninger [98], Wimber, and Van Cott [99] and Kim et al. [100]. From 2009 to 2021, there were nine studies on polyploidy induction using antimitotic agents in *Cymbidium*, of which eight were performed with hybrid cultivars (Table 3).

Similar to *Cattleya*, in *Cymbidium*, most polyploid induction studies were performed under in vitro conditions, and the PLBs were the primary type of explants (55.6%). Other explants included were protocorms, rhizomes, seedlings, and young shoots (Table 3).

The rates of obtaining polyploid plants ranged from 11.1% to 60%, and colchicine was used as an antimitotic in 89% of the studies and oryzalin in only two studies. The highest regeneration rates were obtained with colchicine in 0.03–0.05% concentrations and drug exposure times ranging from 4 to 7 days (Table 3). The use of oryzalin in two *Cymbidium* hybrids made it possible to obtain tetraploids at concentrations of 5–10 mg L^−1^. However, colchicine was more efficient than oryzalin in PLB survival and polyploid frequency [101]. Another fact reported by these authors was the strong genotype-dependent response, and up to 60% of polyploids were reported in *Cym*. Show Girls, while, in *Cym*. Mystery Island, maximally 16.7% of the polyploids were obtained.

### 5.3. Dendrobium Genus

Similar to other genera, most polyploidy induction studies have been carried out under in vitro culture environments. The induction of polyploidy under ex vitro conditions was performed by Vichiato et al. [93] on *Den. nobile* by the immersion of plants and seedlings in a solution of colchicine at 10 mg L^−1^ for 96 h, which resulted in 29.17% being tetraploid plants. However, these polyploid plants demonstrated a slower and reduced vigorous development concerning those of diploids.

Colchicine was the main antimitotic agent used for the artificial induction of polyploidy in *Dendrobium.* The concentrations of colchicine ranged from 0.01 to 0.2%, and the treatment time ranged from 1 to 14 days of antimitotic exposure. The main explants chosen for the induction of polyploidy in vitro in *Dendrobium* were the PLBs and protocorms.

About 75% and 80% of the polyploids were obtained at concentrations of 0.2% of colchicine exposed for two days and 0.05% of colchicine for three days, respectively, using the PLBs as explants [108,109] (Table 4). In addition, three studies used oryzalin, propyzamide, and AMP for polyplodization (Table 4). In addition to oryzalin [110], propyzamide at 100 µM for two days [111] and amiprofos-methyl (AMP) at concentrations of 10 mg L^−1^ for 12 to 48 h [112] showed good efficiency in obtaining *Dendrobium* polyploids.

### 5.4. Phalaenopsis Genus

Griesbach [20] was one of the pioneer used in vitro tools for artificial induction of chromosomal polyploidization in *Phalaenopsis*. Protocorms of *Phal. Equestris*, *Phal. Fasciata*, and *Phal*. “Betty Hausermann” were exposed to 50 mg L^−1^ of colchicine for ten days, resulting in 46% with polyploid seedlings [20]. Griesbach [121] used a similar technique with a colchicine treatment to restore the fertility of the triploid hybrid *Phal*. Golden Sands “Canary” and obtained 50% of hexaploid plants (fertile), which were successfully used as parental plants to develop new cultivars with a greater intensity of colors, sizes, and shapes, such as the pentaploid (2n = 5x = 95) *Phal*. Meadowlark [121].

Twelve polyploid induction studies were reported for the genus *Phalaenopsis*, and colchicine was used in 80% of these studies in concentrations ranging from 0.5 to 5000 mg L^−1^. The colchicine exposure time lasted from 3 to 10 days. Oryzalin was applied only in one study (Table 5).

The main explants chosen for the in vitro induction of polyploids are protocorms [20,30,121,122]. On the other hand, the explants chosen for the ex vitro induction are seedlings and flowers (bud flowers and pollinated flowers) [123,124,125] (Table 5). Furthermore, it was observed that, unlike other genera, in *Phalaenopsis*, 50% of the studies in the literature used organs or entire plants exposed to colchicine under ex vitro conditions to obtain polyploid plants.

Interestingly, several studies were performed on *Phal*. *amabilis* (64%) during 2013–2021 (Table 5). Despite the reduced commercial importance of this species compared to the hybrids, it has been extensively studied under in vitro culture conditions and for different purposes, possibly serving as an in vitro regeneration model among the many *Phalaenopsis* genotypes. Among these studies, we would like to highlight the treatment of nitrous oxide to pollen grains of *Phal.s amabilis* (2n = 2x = 38), which was used to obtain seeds and seedlings from the treated pollen grains. In which the treatment for 24 h resulted in up to 35.6% triploid and 6.7% tetraploid plants, which was more efficient than the treatment for 48 h. Furthermore, Azmi et al. [123] obtained up to 100% tetraploid plants of *Phal. amabilis* using colchicine (0 to 2000 mg L^−1^) soaked in wet cotton covered with aluminum foil and applied to the ovaries and stigma three days after self-pollination.

### 5.5. Induction of Polyploidy in Oncidium, Vanda, and Others

There have been a few reports on the artificial induction of polyploidy in the genus *Oncidium*. Unemoto et al. [130] observed that an increased exposure time of protocorms of *Onc. flexuosum* to colchicine resulted in the increased death of explants, and the surviving seedlings demonstrated morphological alterations with a reduction of shoots and roots. However, the authors did not analyze the ploidy level of the regenerated plants. Therefore, it is difficult to tell if these changes were due to the colchicine phytotoxicity or polyploidization of the regenerated plants. Similarly, Cui et al. [131] also observed morphological changes such as smaller and more robust plants, thick leaves, and longer stomata lengths associated with polyploids, compared to the untreated diploid plants of nonidentified/specified *Oncidium* obtained from thin cell layers of PLBs treated with different concentrations and exposure times of colchicine.

Nakasone [132] was the first to induce polyploidy in *Vanda* “Miss Joaquín” using young shoots treated with different concentrations of colchicine (0.5% and 1.5%) for 2 and 6 days of exposure (Table 6). More recently, Tuwo and Indrianto [133] obtained polyploid plants from protocorms treated with colchicine (0.5% for 6 h) for the hybrid of *V.a limbiata* Blume X *V. tricolor* Lindl. var. *suavis*. The regenerated polyploid plants presented a smaller number and width of leaves, smaller number and length of roots, and a greater stomatic size and lower stomatal index (Table 6).

There have been two studies on the genus *Rhynchostylis*, correlated with *Vanda*, in which colchicine was applied to PLBs in one study [134]. The herbicide propyzamide was applied to seeds of two genotypes of this genus to induce polyploidy [111].

In addition to these, studies with the genera *Calanthe*, *Epidendrum*, *Odontioda*, and *Paphiopedilum* were also reported (Table 6).

## 6. Conclusions

The high frequency of endopolyploidy, together with the presence of polysomatic organs and tissues, were observed in different orchid genera. In vitro regeneration pathways, such as PLBs induction and regeneration from tissues with a high frequency of endoreduplication, can be used to obtain polyploid plants without antimitotic treatment. Additionally, the development of unreduced gametes was reported in some species of Orchidaceae, which is a natural mechanism of polyploidization. These genotypes were used as parents in breeding programs. The use of antimitotic agents is an efficient technique for the artificial production of polyploid plants, which increases the number of genotypes with useful ornamental characteristics in the world flower industry. The genera *Cymbidium*, *Dendrobium*, and *Phalaenopsis*, with the most significant impact on the world’s floriculture, have the highest number of published studies and reports on obtaining polyploid plants. In vitro cultivation, using protocorms and PLBs as explants and colchicine as an antimitotic agent has most widely been used for the artificial induction of polyploids in orchids.

## 7. Further Prospects

Although colchicine is widely used to increase the frequency of polyploids in orchids, most studies have evaluated the effectiveness of its concentration and exposure. Furthermore, few studies have been focused on assessing the conditions of its application on the explants. The different exposure times, treatment temperatures, and joint applications of products that increase the absorption of colchicine by tissues or reduce its toxicity needs to be better understood. Most studies have reported a pronounced effect of this reagent on the survival of treated explants and different symptoms associated with phytotoxicity on tissues, with biochemical changes and plantlet development after treatment. However, practically no studies have been reported to alleviate the phytotoxic effects of this reagent on explant development, which are limited by successive washing with distilled or deionized water. Another relevant fact of using colchicine, besides its high cost, is toxicity to humans and animals, resulting in short-, medium-, and long-term effects [138]. Thus, the generation of more natural polyploid plants by promoting the formation of new PLBs from polysomatic tissues or by increase studies with unreduced gametes are exciting strategies with lower risks than colchicine induction.

The correlation between polyploidy and genetic improvement is remarkable. In some genera, like *Phalaenopsis* [36], polyploid commercial hybrids are predominately used in floriculture.

Although most *Phalaenopsis* hybrid cultivars are protected in terms of commercialization, a strategy that could be further explored by breeding companies would be the use of triploid and pentaploid cultivars, which also have limitations in terms of sexual reproduction.

Another relevant fact is the lack of studies that contemplate haploid and double-haploid plant technology in orchids, a technology currently used for large crops with diverse breeding and genetic applications.

## Figures and Tables

**Table 2:** Polyploidy induction of *Cattleya* using antimitotic agents.

Species/ Cultivar	Type of Explant	Treatment	Polyploidy Assessment Method	Polyploidy Efficiency	Anatomical and Morphological Features of Polyploids	References
*Cattleya intermedia Lind.*	PLBs	Colchicine 0.05% for 4 days for Clon 114 Colchicine 0.1% for 4 days for Clon 121	Chromosome count Stomatal density	Clon 114 Mixoploides (42.0%) Tetraploides (29.0%) Clon 121 Mixoploides (33.0%) Tetraploides (22.0%)	Higher stomatal density	Silva, Callegari-Jacques, and Bodanese-Zanettini [96]
*Cattleya tigrina Lind.*	Plantlets	Colchicine 12.5 mM for 48-h	Flow cytometry Stomatal density Stomatal functionality Stomatal index	72.5% polyploids	Greater stomatal functionality, lower stomatal density, lower stomatal index	Menezes-Sá et al. [97]

PLBs: Protocorm-like bodies.

**Table 3:** Polyploidy induction of *Cymbidium* using antimitotic agents.

Species/Cultivar	Type of Explant	Treatment	Polyploidy Assessment Method	Polyploidy Efficiency	Anatomical and Morphological Features of Polyploids	References
*Cymbidium* ‘Promised Land’	PLBs	Oryzalin 14.4, 28.9 and 57.7_M	-	-	-	Miguel and Leonhardt [30]
*Cym. hydridum*	PLBs	Colchicine 0.1% for 3 days	Chromosome count Morphological characterization	27.6% polyploids	Shorter length and number of roots, larger root diameter	Xie et al. [43]
Hybrids: *Cym.* Showgirls “Sirly” and *Cym.* Mystery Island “Silk Road”	PLBs	Colchicine 50 mg L^−1^ for 7 days Oryzalin 5 mg L^−1^ for 2 weeks Colchicine 50 mg L^−1^ for 7 days Oryzalin 10 mg L^−1^ for 3 days	Flow cytometry Morphological characterization Anatomical characterization	*Cymbidium* Showgirls “Sirly” (60.0% with colchicine and 47.0% with oryzalin) *Cymbidium* Mystery Island “Silk Road” (16.7% with colchicine and 6.7% with oryzalin)	Shorter and wider dark green leaves, less growth. Greater length and width of guard cells, lower stomatal density.	Hwang, Kim, and Park [101]
*Cym.* Ruby Shower ‘Murasakin Okimi’	PLBs	Colchicine 300 mg L^−1^ for 15 days	Chromosome count	30.0% polyploids		Yang et al. [102]
*Cym. hybridum*	PLBs	Colchicine 0.05% for 5 days	Morphological characterization Stomatal density Anatomical characterization	23.7% polyploids	Darker, compact and resistant leaves	Wang et al. [103]
*Cym. lowianum*	Seedlings	Colchicine 0.04% for 72 h	Morphological characterization Anatomical characterization	60.0% polyploids	Short stem, obscure leaves, greater width, less growth. Larger size of stomata, smaller number of stomata	Xuejiao, Zhilin, and Lipin [104]
*Cym. hybridum*	Young shoots	Colchicine 0.05% por 24 h	Morphological characterization Anatomical characterization	28.2% polyploids		Ji et al. [105]
*Cym. sinense* ‘QiJianBaiMo’	Rhizome	Colchicine 0.01% for 3 days		11.1% polyploids	Hard leaves and thickened roots	Mugui et al. [106]
Hybrid: *Cym. sinenthese* ‘Lv mosu’ × *Cym. hybridum* ‘Shijieheping’	Protocorms	Colchicine 0.03% for 72 h	Flow cytometry Morphological characterization Anatomical characterization	36.0% polyploids	Wider green leaves, thicker roots and less growth. Greater length and width of guard cells, lower stomatal density.	Song et al. [107]

PLBs: Protocorm like-bodies.

**Table 4:** Polyploidy induction of *Dendrobium* using antimitotic agents.

Species	Types of Explant	Treatment	Polyploidy Assessment Method	Polyploidy Efficiency	Anatomical and Morphological Features of Polyploids	References
*Dendrobium* **‘Gatton Sun** **Ray’**	PLBs	Oryzalin 14.4 μM (5 mg L^−1^) for 6 days	Anatomical characterization		Longer stomata length	Miguel and Leonhardt [30]
*Den. nobile*	Seedlings	Colchicine 0.1% for 96 h	Chromosome count Morphological characterization	29.2% polyploids	Smaller height, smaller diameter of pseudobulbs, smaller leaf length, larger leaf width.	Vichiato et al. [93]
*Den. phalaenopsis* × *Den. loddigesii*	PLBs	Colchicine 0.05% (5 mg L^−1^) for 3 days	Flow cytometry Anatomical characterization	80.0% polyploids	Longer stomata length, lower stomatal density.	Grosso et al. [108]
*Den. formosum*	PLBs	Colchicine 0.2% (20 mg L^−1^) for 48 h	Morphological characterization Anatomical characterization Stomatal density	75.0% polyploids	Longer stem, thick green leaves—obscure. Larger stomata size, lower stomatal density.	Yenchon and Te-chato [109]
*Den. officinale*	Protocorms	Oryzalin 14.4 μM (5 mg L^−1^) for 24 h	Flow cytometry Chromosome count	37.4% polyploids	Smaller height, smaller leaf length, smaller root length, larger stem and root diameter, larger lip and gynostemium width.	Zhang and Gao [110]
*Den.* **‘Burana Jade’**	Protocorms	Pronamide 100 μM for 2 days	Morphological characterization Anatomical characterization	33.3% polyploids	Thickened leaves, shorter length, short and thickened stem. Greater number of stomata.	Tantasawat et al. [111]
*Den.* stardust “Fire Bird”	PLBs	Amiprofosfo-metil (AMP) 160 mg for 12	Flow cytometry Chromosome count Morphological characterization	80.0% amphydiploids	Larger stem size and number of leaves.	Kondo et al. [112]
*Den. phalaenopsis*	Protocorms	Colchicine 0.05% for 9 days	Chromosome count Morphological characterization	50.0% polyploids	Thick and dark green leaves, greater number of flowers/inflorescences. Greater guard cell length, greater guard cell width.	Chaicharoen and Saejew [113]
*Den. secundum*	Protocorms	Colchicine 0.05% for 1 day	Flow cytometry Chlorophyll Content Anatomical characterization		Greater thickness of roots, stem and leaves, greater flower size. Greater length of guard cells.	Atichart and Bunnag [114]
*Den. srabrilingue*	PLBs	Colchicine 0.075% (7.5 mg L^−1^) for 14 days	Flow cytometry	43.1% polyploids	Larger diameter of stem and roots, dark green leaves.	Sarathum et al. [115]
*Den.* “Miss Singapore”	Protocorms	Colchicine 0.01% (1 mg L^−1^) for 2 days	Flow cytometry Chromosome count			Bunnag and Hongthongkham [116]
*Den. chrysotoxum*	PLBs	Colchicine 0.04% (4 mg L^−1^) for 24 h	Flow cytometry	47.0% tetraploids		Atichart [117]
*Den. draconis*	Protocorms	Colchicine 0.05% (5 mg L^−1^) for 3 days	Flow cytometry Morphological characterization Anatomical characterization	43.0% polyploids	Smaller stem size; large, thick, dark green leaves. Smaller guard cell number/area unit, larger guard cell size.	Bunnag and Hongthongkham [118]
*Den.* ‘Sonia’	PLBs	Colchicine 0.15% (15 mg L^−1^) for 3 days	Flow cytometry Chromosome count Morphological characterization Anatomical characterization	26.6% polyploids	Greater mass and smaller width of the bulb, greater length and width of leaves. Longer stomata length.	Zakizadeh, Kaviani, and Hashemabadi [119]
*Den. cariniferum*	Protocorms	Colchicine 0.05% (5 mg L^−1^) for 24 h	Flow cytometry Morphological characterization Anatomical characterization	33.0% polyploids	Greater width and thickening of leaves, greater diameter of stem and root. Greater length of stomata, lower stomatal density, greater number of chloroplasts, thickened spongy tissue, larger leaf veins, smaller cells of the adaxial epidermis and trichomes	Zhang and Gao [120]

PLBs: Protocorm-like bodies.

**Table 5:** Polyploidy induction of *Phalaenopsis* orchids.

Species	Type of Explant	Treatment	Polyploidy Assessment Method	Polyploidy Efficiency	Anatomical and Morphological Features of Polyploids	References
*Phal. equestris*, *Phal. fasciata*, *Phal.* Betty Hausermann	Protocorms	Colchicine 50 mg L^−1^ for 10 days	Chromosome count	46.0% polyploids		Griesbach [20]
*Phal. bellina*	Protocorms	Oryzalin 14.4 μM (5 mg L^−1^) for 3 days	Anatomical characterization		Longer stomata length	Miguel and Leonhardt [30]
*Phal. amabilis* var. *grandiflora*	In vitro plantlets from PLBs	0.15% colchicine for 72 h under bubble bioreactor	Flow cytometry, morphological and cytological measurements		Reduction in plantlet length and number, and stomatal density; Increases in leaf number and width, guard cells and chloroplast number	Mohammadi, Kaviani, and Sedaghathoor [41]
*Phal*. Goden Sands “Canary”	Protocorms	Colchicine 0.5 mg L^−1^ for 10 days		50.0% polyploids	More obscure flowers, larger and larger in diameter	Griesbach [121]
*Phal. amabilis*; *Phal. amboinensis*	Protocorms	Colchicine 50 mg L^−1^ for 10 days	Chromosome count Anatomical characterization Stomatal density	33.3% polyploids of *Phal. amabilis* 40.0% polyploids of *Phal. amboinensis*	Thick dark green leaves. Longer stomatal length, lower stomatal density	Rahayu et al. [122]
*Phal. amabilis*	Pollinated flowers	Colchicine 50 mg L^−1^ for 3 or 5 days Colchicine 500 mg L^−1^ for 5 days	Morphological characterization Anatomical characterization	60.0% polyploids 100.0% polyploids	Shorter leaf length, shorter length and larger diameter of roots, longer length and diameter of the basal organ of the protocorm. Longer stomatal length	Azmi et al. [123]
*Phal. amabilis*	Bud flowers	Colchicine for 3 days at 50, 500 or 1000mg L^−1^	Morphological characterization	71.2% polyploids * 86.7% polyploids * 100.0% polyploids *	Smaller root size, larger number of roots, larger root thickness, larger stem diameter, larger length of the basal organ of the protocorm.	Azmi et al. [124]
*Phal. amabilis*	Seedlings	Colchicine 5000 mg/L	Morphological characterization Anatomical characterization Stomatal density	50.0% polyploids	Greatest height Greater length and width of stomata, lower stomatal density	Rahayu et al. [125]
*Phalaenopsis*	PLBs	Colchicine 500, 1000 and 2000 mg L^−1^ for 1, 3 e 7 days				Cui G. [126]
*Phal. amabilis*	Pollen grains	Nitrous oxide (N_2_O) for 24 h Nitrous oxide (N_2_O) for 48 h	Chromosome count Flow cytometry	Triploids (36.0%) Tetraploids (7.0%) Tetraploids (5.0%)	-	Wongprichachan et al. [127]
*Phal. pulcherrima* (ex *Doritis pulcherrima*)	Protocorms	Colchicine 100 mg L^−1^ for 10 days	Chromosome count	25.0% polyploids		Rungruchkanont and Apisitwanich [128]
*Phal. amabilis*	Auxiliary gems	Colchicine 0.2% for 48 h	Chromosome count Flow cytometry Anatomical characterization Morphological characterization	70.0% polyploids	Increase in size and decrease in density of guard cells	Zaker Tavallaie and Kolahi [129]

* Putative polyploids; PLBs: Protocorm-like bodies.

**Table 6:** Other genus of *Orchidaceae* with at last one study with an induction of polyploids.

Species	Explant	Treatment	Polyploidy Assessment Method	Polyploidy Efficiency	Anatomical and Morphological Features of Polyploids	References
*Epidendrum* **‘Helen’s Pride’**	Protocorms	Oryzalin 57.7 μM (20 mg L^−1^) for 6 days	Anatomical characterization	2 polyploids	Longer stomata length	Miguel and Leonhard [30]
*Odontioda* **‘Emma Sander’**	Protocorms	Oryzalin 28.9 μM (10 mg L^−1^), 14,4 μM (5 mg L^−1^) or 57.7 μM (20 mg L^−1^) for 6 dias	Anatomical characterization	3 polyploids 2 polyploids 3 polyploids	Longer stomata length	Miguel and Leonhardt [30]
*Rynchostylis gigantea* ‘K3.0124W’ *Rynchostylis gigantea* ‘K3.0131W’	Seeds	Pronamide 200 μM for 4 days	Chromosome count Morphological characterization Stomatal density Anatomical characterization	*Rynchostylis gigantea* ‘K3.0124W’ (23.0%) *Rynchostylis gigantea* ‘K3.0131W’ (35.0%)	Thick, rounded leaves. Anatomic: lower stomatal density, larger stomata size.	Tantasawat et al. [104]
*Vanda* “Miss Joaquin”	Young shoots	Colchicine 0.5% (50 mg L^−1^) and 1.5% (150 mg L^−1^) for 6 days	Chromosome count Anatomical characterization		Longer stomatal length	Nakasone [132]
Hybrid *Vanda limbiata* **Blume** X *Vanda tricolor* Lindl. var. *suavis*	Protocorms	Colchicine 0.5% (50 mg L^−1^) for 6 h	Flow cytometry Chromosome count Morphological characterization Anatomical characterization		Smaller number of roots, smaller root length, smaller number of leaves, smaller leaf width. Anatomical: Larger stomatal size, smaller stomatal index.	Tuwo and Indrianto [133]
*Rynchostylis gigantea* var. *rubrum sagarik*	PLBs	Colchicine 20 mg L^−1^ for 72 h	Chromosome count Morphological characterization Stomatal density Number of chloroplasts	60.0% polyploids	Smaller size of seedlings and roots, larger number of leaves, smaller leaf size. Higher stomatal density, lower number of chloroplasts	Kerdsuwan and Te-chato [134]
Hybrid *Calanthe discolor* X *Calanthe sieboldii*	Seeds	Colchicine 0.1% (10 mg L^−1^) for 7 days Oryzalin 0.003% (0,3 mg L^−1^) for 1 days	Flow cytometry Size of stomata Stomatal density	81.0% polyploids 86.0% poliploids	Smaller size, thick dark green rounded leaves, larger leaf width, larger stem and root diameter. Lower stomatal density, larger stomatal size	Chung et al. [135]
*Paphiopedilum villosum*	Sprouts	Colchicine 50 μM (20 mg L^−1^) for 6 days	Flow cytometry Chromosome count Anatomical Characterization Stomatal density	19.9% polyploids	Greater leaf length, greater leaf width. Longer guard cell length, lower stomatal density	Huy et al. [136]
*Paphiopedilum callosum*	Seeds	Colchicine 1000 mg L^−1^ for 54 h	Chromosome count Morphological characterization Anatomical characterization		Larger seedling size	Suhaila Siti et al. [137]

PLBs: Protocorm-like bodies.

## Data Availability

All tables and figures are original from authors.
